# Cold-Chain Adaptability During Introduction of Inactivated Polio Vaccine in Bangladesh, 2015

**DOI:** 10.1093/infdis/jiw591

**Published:** 2017-07-01

**Authors:** Mallick M. Billah, K. Zaman, Concepcion F. Estivariz, Cynthia J. Snider, Abhijeet Anand, Lee M. Hampton, Tajul I. A. Bari, Kevin L. Russell, Shua J. Chai

**Affiliations:** 1 Field Epidemiology Training Program Bangladesh, Institute of Epidemiology, Disease Control and Research, Dhaka, Bangladesh,; 2 Expanded Program for Immunization, Ministry of Health and Family Welfare, Dhaka, Bangladesh, and; 3 icddr,b (formerly International Centre for Diarrhoeal Diseases Research, Bangladesh), Dhaka, Bangladesh;; 4 Global Immunization Division and; 5 Division of Global Health Protection, Center for Global Health, Centers for Disease Control and Prevention, Atlanta, Georgia

**Keywords:** polio, routine immunization, inactivated polio vaccine, new vaccine introduction, cold chain, vaccine transportation, freezing.

## Abstract

**Background.:**

Introduction of inactivated polio vaccine creates challenges in maintaining the cold chain for vaccine storage and distribution.

**Methods.:**

We evaluated the cold chain in 23 health facilities and 36 outreach vaccination sessions in 8 districts and cities of Bangladesh, using purposive sampling during August–October 2015. We interviewed immunization and cold-chain staff, assessed equipment, and recorded temperatures during vaccine storage and transportation.

**Results.:**

All health facilities had functioning refrigerators, and 96% had freezers. Temperature monitors were observed in all refrigerators and freezers but in only 14 of 66 vaccine transporters (21%). Recorders detected temperatures >8°C for >60 minutes in 5 of 23 refrigerators (22%), 3 of 6 cold boxes (50%) transporting vaccines from national to subnational depots, and 8 of 48 vaccine carriers (17%) used in outreach vaccination sites. Temperatures <2°C were detected in 4 of 19 cold boxes (21%) transporting vaccine from subnational depots to health facilities and 14 of 48 vaccine carriers (29%).

**Conclusions.:**

Bangladesh has substantial cold-chain storage and transportation capacity after inactivated polio vaccine introduction, but temperature fluctuations during vaccine transport could cause vaccine potency loss that could go undetected. Bangladesh and other countries should strive to ensure consistent and sufficient cold-chain storage and monitor the cold chain during vaccine transportation at all levels.

Following the polio endgame strategy suggested by the Global Polio Eradication Initiative [1, 2], Bangladesh introduced 1 dose of inactivated poliovirus vaccine (IPV) in the routine immunization schedule [3] in March 2015 and concurrently switched routine immunization with trivalent to bivalent oral poliovirus vaccine (OPV) (ie, removing type 2 poliovirus serotype) in April 2016. Because a country’s routine immunization program requires an effective cold chain for maintaining optimum potency of vaccines [4, 5], introduction of IPV (or any new vaccine) can create challenges for cold-chain storage capacity [6] and for maintaining optimum temperatures during vaccine transport. 

At optimal temperatures (2°C–8°C), IPV can be stored up to 2 years; temperatures outside this range may reduce vaccine potency [7]. Potential damage because of exposure to high temperatures can be detected through the presence of vaccine vial monitors (VVMs) on all IPV vials [8]. VVMs are specialized vial labels that provide a visual indication of exposure to elevated temperatures for a period of time sufficient to alter vaccine potency, which for IPV is 45 days at 25°C. However, there is no mechanism for detecting exposures to freezing temperatures. The “shake test,” which consists of shaking a vaccine vial suspected of exposure to freezing temperatures and comparing its sedimentation rate with a vial that has not been frozen, cannot be used for IPV because of its chemical composition [6].

In Bangladesh, the Expanded Program on Immunization (EPI) of the Ministry of Health and Family Welfare (MOHFW) imports and stores vaccines at a national depot. At subnational levels, management for vaccine storage and delivery of immunization services varies. In districts, which consist of rural areas and small cities, vaccines are managed and delivered by staff from the MOHFW directly under the EPI. From the national depot, vaccines are delivered every 3 months to district depots, and from district depots vaccines are delivered monthly to government and nongovernment health facilities at the subdistrict and ward (below subdistrict) levels using cold boxes. In large cities (referred to below as *city corporations*), vaccines are managed by nongovernmental organizations under the authority of the Ministry of Local Government Rural Development and Cooperatives [9].

Vaccines are delivered every 3 months from national depots to city corporation depots. From city corporation depots, vaccines are distributed monthly to nongovernment health facilities using cold boxes. In all health facilities (government or nongovernment; catchment of about 300 000–400000 persons each, on average) [9], vaccines are delivered in immunization sessions on site or are transported in vaccine carriers to a distribution point, where they are given to vaccinators for use at outreach vaccination sessions, each of which cover, on average, 1000 persons [9]. Carriers are used to store vaccines during sessions and returned to facilities with unused vaccines daily at the end of the session (Figure 1). Along with IPV, vaccine carriers and cold boxes transport other vaccines administered in routine EPI, such as bacille Calmette-Guerin (BCG) and pentavalent (diphtheria, tetanus, pertussis, hepatitis B and *Haemophilus influenzae* type b) vaccines [9].

**Figure 1.**
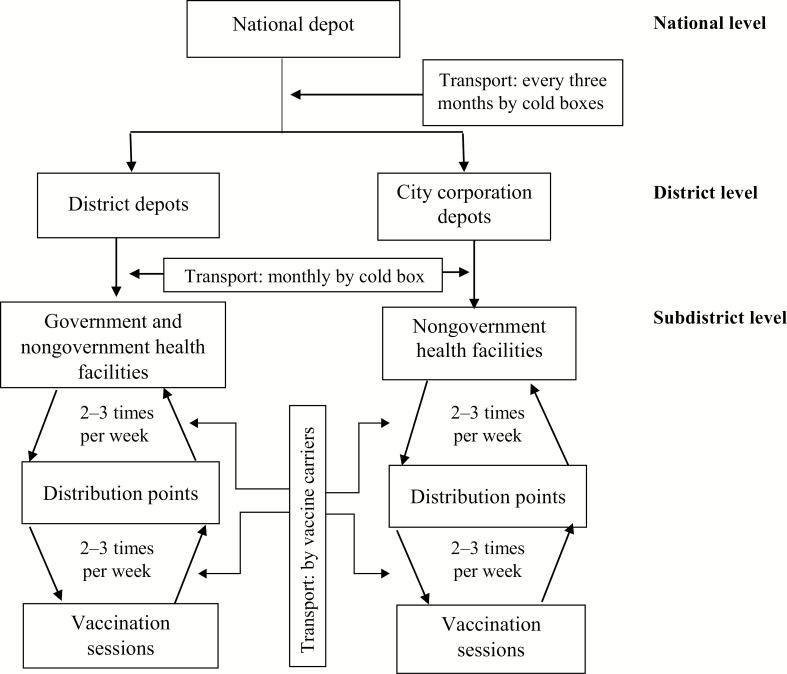
Cold-chain distribution of vaccines in Bangladesh, 2015.

In preparation for IPV introduction, Bangladesh’s EPI assessed the space requirements and trained the cold-chain managers, supervisors, and vaccinators, with technical assistance from the World Health Organization (WHO). Because Bangladesh was among the first OPV-providing countries to introduce IPV in routine immunization, we conducted a cross-sectional assessment of cold-chain capacity and transport for vaccines 5 months after the introduction of IPV, to understand the successes and lessons learned for IPV delivery.

## METHODS

### Study Locations and Study Period

We selected 5 districts and 3 city corporations from each of 8 administrative divisions across Bangladesh by convenience to include sites with different routine immunization program management, pentavalent vaccination coverage (because IPV was scheduled with the third dose of pentavalent vaccine), and levels of access to immunization [10]. Districts were stratified into urban, rural, and areas with low access to immunization, and 1 ward was selected from each stratum. In city corporations, we randomly selected 3 wards. Among the 24 selected wards, we collected data on cold-chain capacity and management in 23 health facilities (14 district and 9 city corporation health facilities) that stored and supplied vaccines for vaccination sites (1 facility supplied vaccines for 2 wards), and in 1 or 2 outreach vaccination sites per ward (n = 48). Data collection occurred during August–October 2015.

### Data Collection

Five field teams collected cold-chain storage and vaccine transport data using a modified version of WHO’s postintroduction evaluation tool [11]. The study team trained field teams on study objectives and procedures, including skills in interviewing. Data collection instruments and procedures for temperature monitoring were pilot tested and revised before use in the field. Field teams interviewed immunization and cold-chain managers at health facilities and vaccinators at outreach vaccination sites, and observed cold-chain equipment and practices using a standardized checklist. During the interviews and observations, the teams collected information on cold-chain storage capacity and functionality. Because electricity outages can last from a few minutes to hours in Bangladesh, teams obtained the history of power failures, the use of standard protocols in the event of power failures, and the availability of alternate power sources and voltage stabilizers, which protect equipment against damaging voltage fluctuations. Teams examined temperature monitoring practices, such as maintenance of temperature logs in freezers and refrigerators, and use of temperature monitoring devices during vaccine transport. Teams also checked for the presence of expired or frozen vaccine vials or vials with VVMs in stage 3–4 (not usable) in cold-chain storage.

The field teams (or trained cold-chain managers) placed LogTag recorders (Model TRIX-8; accuracy, ±0.5°C for −20°C to +40°C) [12] to record temperatures at 5-minute intervals for 5–6 hours inside 1 ice-lined refrigerator (ILR) used for vaccine storage in each of the district and city corporation depots (n = 8) and health facilities (n = 23). LogTag recorders were also placed in cold boxes used to transport vaccines from district and city corporation depots to health facilities (n = 19) and in 2 vaccine carriers sent to each outreach vaccination site (n = 48). Recorders were placed in vaccine carriers together with vaccines before transport of vaccines for the vaccination session and were removed when carriers were returned to the facility with leftover vaccines at the end of the vaccination session. The LogTag recorders were procured specifically for this study and are not used routinely by the Bangladesh EPI to monitor temperatures during vaccine transportation.

### Data Entry and Analysis

Team members entered the information collected on paper-based questionnaires and checklists daily into a Microsoft Office Access 2007 database or a Microsoft Excel 2013 spreadsheet. On receipt of a used LogTag recorder, teams downloaded temperature data using the LogTag Analyzer Software [12]. Temperature monitoring data and paper-based transport event data were linked by location, date, and time of placement of the LogTag recorders in Excel. Because LogTag recorders required 40 minutes to equilibrate after placement, temperature data for the first 40 minutes were excluded from the analysis. Per EPI cold-chain guidelines, all vaccines (except OPV) should be stored and transported at temperatures between 2°C and 8°C. The temperatures measured in cold-chain equipment were categorized into 3 groups: within optimum (2°C–8°C), above optimum (>8°C), and below optimum (<2°C) range. We considered temperature deviations critical if vaccines had been continuously exposed to temperatures outside the optimum range for >60 minutes.

We performed descriptive analyses using Microsoft Excel 2013 and Stata v13.0 (StataCorp, College Station, TX) software. Because a mix of nonprobability and probability sampling was used to identify areas for inclusion in the study, we did not calculate confidence intervals around proportions or conduct statistical testing.

### Ethical Considerations

Human subjects review by the US Centers for Disease Control and Prevention (CDC) determined this project to be a public health program evaluation that was not human subjects research. The protocol was approved by the Ethical Review Committee of icddr,b. Permission from the EPI and MOHFW of Bangladesh was obtained for the evaluation of the cold chain.

## RESULTS

### Cold-Chain Capacity and Maintenance

Based on observations of cold-chain storage capacity, nearly all district and city corporation depots (≥88%) and health facility storage sites (≥87%) had a separate room for vaccines, kept them in an organized way, and had no other items in the refrigerators other than vaccines. Essentially all district and city corporation depots and health facility storage sites (≥96%) had the required functioning equipment for cold-chain storage, including ILRs and cold boxes. A freezer to store ice packs and vaccines that required freezing was available in most (≥88%) of the district and city corporation depots and health facilities (Table 1).

**Table 1.** Characteristics of Cold-Chain Equipment in District and City Corporation Depots and Health Facilities Observed After Introduction of IPV and PCV in Bangladesh, 2015

Characteristic	No. (%)	
	Districts and City Corporations (n = 8)	Health Facilities (n = 23)
Cold-chain room and equipment		
Absence of materials other than vaccine in refrigerators (eg, food, drinks, other drugs)	8 (100)	23 (100)
Presence of a separated and dedicated room for cold chain	8 (100)	20 (87)
Vaccines, diluents, syringes kept in a well-organized way	7 (88)	22 (96)
Facilities with clean, functioning equipment		
Ice-lined refrigerators	8 (100)	23 (100)
Cold boxes	8 (100)	22 (96)
Freezers	7 (88)	22 (96)
Refrigerators	2 (25)	4 (17)
Facilities with equipment not clean or not functioning	1 (13)	3 (13)
Power supply		
Presence of flowchart or protocol for response to power failure	7 (88)	23 (100)
Presence of voltage stabilizer connecting cold-chain equipment	3 (38)	10 (44)
Presence of alternative power source (eg. solar)	2 (25)	3 (13)
Power interruptions >24 h in last 3 mo	1 (13)	3 (13)
Temperature monitoring		
Presence of functional temperature monitoring devices	8 (100)	23 (100)
Temperature charts up to date	8 (100)	23 (100)
Temperature recorded twice daily	8 (100)	23 (100)
Temperatures recorded on weekend/holidays	3 (38)	14 (61)
Temperature charts showing nonoptimal temperatures (<2^o^C or >8^o^C) >5 times in last 1 mo	2 (25)	6 (26)
Use of thermometers during vaccine transportation		
From district and city corporation depots to health facilities	3 (38)	…
From health facilities to outreach vaccination sites	…	5 (21)
Changes in cold storage to accommodate IPV and PCV reported by immunization manager		
Increased storage	5 (63)	3 (13)
Did not change and was sufficient	3 (37)	12 (52)
Did not change and was not sufficient	0 (0)	8 (35)

Abbreviations: IPV, inactivated polio vaccine; PCV, pneumococcal conjugate vaccine.

With the introduction of IPV and pneumococcal conjugate vaccine (PCV), immunization managers of 63% of district and city corporation depots reported that their sites required an increase in, and these sites had increased, their cold-chain storage capacity. In health facilities, however, 35% reported insufficient optimal storage capacity and required additional equipment, although they were still able to support the vaccination program. The equipment needs reported included refrigerators (88%), freezers (38%), cold boxes (25%), and vaccine carriers (25%).

In the 3 months before the interview, 13% of district and city corporation depots and health facilities reported power failures lasting >24 hours. Of these sites, none had a generator or alternative (solar) power source, so vaccines had to be placed in cold boxes or moved to other storage sites, or a generator had to be rented. The team observed a generator or solar power in 2 other district and city corporation depots and 4 other health facilities. Flowcharts or written protocols for responding to power failures were available in all but 1 district depot and all health facilities. The district depot without a protocol for responding to power failures was the same one reporting a power failure lasting >24 hours within the past 3 months. Less than half of district and city corporation depots and health facilities had voltage stabilizers.

All 17 observed vaccine carriers used in outreach sessions in districts were in good condition and contained 4 ice packs inside, but 25% of observed carriers for outreach sessions visited in city corporations had fewer than the required 4 ice packs. The teams did not find IPV vials that were frozen or with VVMs in stage 3 or 4 (requiring disposal because of heat exposure) in any freezer, refrigerator, or vaccine carrier checked during the evaluation.

### Temperature Monitoring Practices in Depots, Health Facilities, and Outreach Vaccination Sites

During their visits, the field teams observed that all 8 district and city corporation depots and 23 health facilities had temperature logs for refrigerators, with temperatures recorded twice daily. However, recording on weekends and holidays only occurred in 38% of district and city corporation depots and 61% of health facilities (Table 1). All storage sites had temperature monitoring devices inside functioning equipment and were recording temperatures at the time of the visit, although the type and number of devices varied by facility and type of cold storage unit (Table 1). Very few (≤ 35%) cold-chain managers of district and city corporation depots and health facilities reported placement of temperature monitoring devices in cold boxes and vaccine carriers during vaccine transport (Table 1). At outreach vaccination sessions, only 17% of vaccine carriers checked contained thermometers to monitor temperature; all 6 were found in vaccination sessions of city corporations, and none in vaccination sessions of districts.

### Measurement of Temperature in Cold-Chain Equipment Using LogTag Recorders

**Figure 2.**
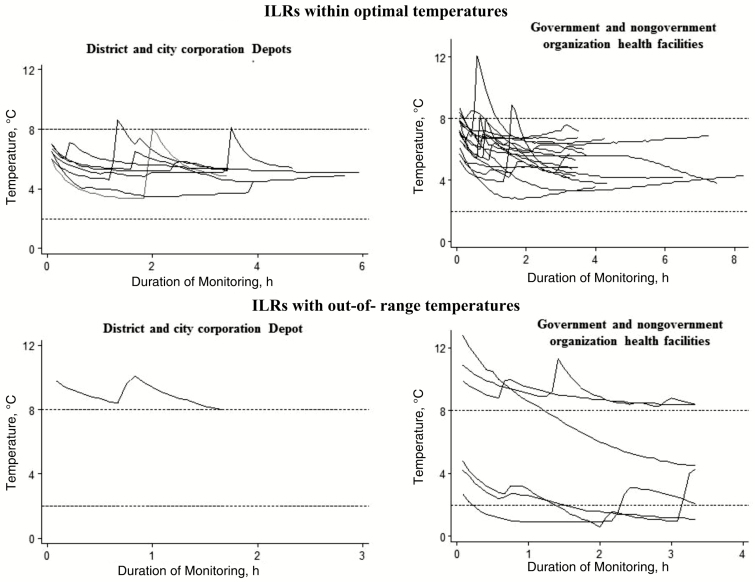
LogTag recorded temperatures of ice-lined refrigerators (ILRs) by site during the study after introduction of inactivated polio vaccine and pneumococcal conjugate vaccine in Bangladesh, 2015.

Among 8 district and city corporation depots, only 1 ILR tested (13%) recorded temperatures >8°C for >60 minutes (Table 2), and no refrigerator reached temperatures <2°C during the recording periods (Table 2 and Figure 2). In health facilities, recorders detected temperatures >8°C for >60 minutes in 13% of ILRs and temperatures <2°C for >60 minutes in 8% (Table 2 and Figure 2).

**Table 2.** LogTag Recording of Cold-Chain Equipment in District and City Corporation Depots and Health Facilities Observed After Introduction of Inactivated Polio Vaccine and Pneumococcal Conjugate Vaccine in Bangladesh, 2015

Cold-Chain Equipment	Total Observed, No.	Temperature Measured, °C		Critical Temperature Excursions (>60 min), No. (%)	
		Minimum	Maximum	<2°C	>8°C
Ice-lined refrigerators					
District and city corporation depots	8	3.4	10.8	0 (0)	1 (13)
Health facilities	23	0.9	12.8	2 (8)	3 (13)
Cold boxes					
National depot to district and city corporation depots	6	3.0	13.4	0 (0)	3 (50)
District and city corporation depots to health facilities	19	−2.1	11.6	7 (37)	1 (5)
Vaccine carriers					
Health facilities in districts	18	1.5	21.0	1 (5)	4 (22)
Health facilities in city corporations	30	0.0	18.7	13 (43)	4 (13)

**Figure 3.**
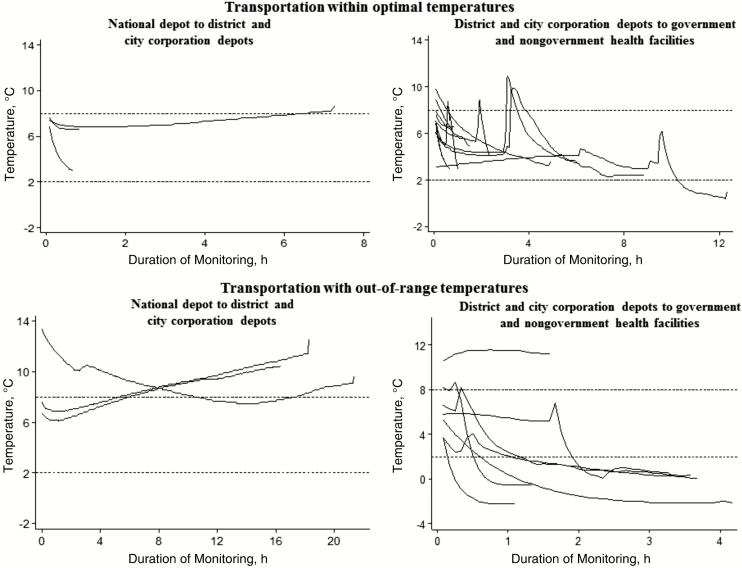
LogTag recorded temperatures of cold boxes transporting vaccines between sites by destination during the study after introduction of inactivated polio vaccine and pneumococcal conjugate vaccine in Bangladesh, 2015.

Half of the LogTag recorders placed in 6 cold boxes used to transport vaccines from the national depot to district and city corporation depots recorded temperatures >8°C for >60 minutes. As shown in Figure 3, the cold boxes that did not maintain correct temperatures were in transport for >5 hours. None reached temperatures <2°C. However, during transport of vaccines between district and city corporation depots and health facilities, 37% of cold boxes reached temperatures <2°C for >60 minutes (Table 2 and Figure 3).

**Figure 4.**
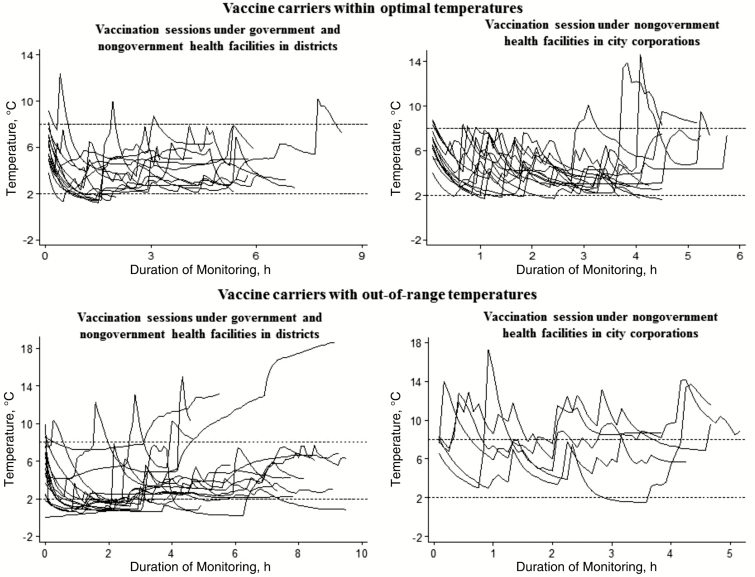
LogTag recorded temperatures of vaccine carriers transporting vaccines to outreach vaccination sessions by type of facilities during the study after introduction of inactivated polio vaccine and pneumococcal conjugate vaccine in Bangladesh, 2015.

For transporting vaccines from health facilities to outreach sessions, recorders detected temperatures <2°C in 5% of observed vaccine carriers for city corporations and 43% of those for districts. Temperatures >8°C were recorded in 22% of vaccine carriers in city corporations and 13% of carriers in districts for up to 14 hours; recorded temperatures reached up to 21°C (Table 2 and Figure 4).

## DISCUSSION

This evaluation of cold-chain capacity found that Bangladesh has substantial cold-chain storage and transportation capacity for vaccines delivered by the routine immunization program after IPV introduction. Key strengths included highly functional storage and transport equipment at higher administrative (national, district, and city corporation) levels and close monitoring of cold storage temperatures at all levels. Areas requiring strengthening included adding alternative power sources and power-surge stabilizers, expanding cold storage capacity at lower administrative (health facility and outreach vaccination sites) levels in some areas, and monitoring of the cold chain during vaccine transport.

Electrical supply from the power grid in Bangladesh can be unstable, and power outages occur regularly [13]. Because district and city corporation depots and health facilities might need to store vaccines for up to 3 months and 1 month, respectively, power failures, especially those that last beyond the recommended 24-hour holdover time for ILRs, can result in wastage of large amounts of vaccines that may lead to serious stock-outs, especially for vaccines with limited supply, including IPV. Nearly all depot sites and health facilities assessed had protocols for responding to power failures—including effective countermeasures, such as shifting vaccines to cold boxes [14]—but these may be insufficient for long-lasting power failures. Use of generators and alternative energy sources are additional steps to provide consistent cold-chain storage that have been shown to be feasible and effective in resource-poor settings [15, 16]. In India, solar energy is used for the cold chain in 18 states, and its use has the additional benefit of supplying power in isolated remote areas [17]. Two district health facilities visited in this study were using solar energy to maintain the cold chain, suggesting that this might be a feasible option for Bangladesh.

Storage capacity is complementary to the need for stable power to ensure that a country’s cold chain can meet the additional storage demand created by introduction of a new vaccine. Cold-chain storage capacity is dependent on vaccination coverage estimates and the physical space needed for vaccines [6]. Based on the assessment of storage capacity, Bangladesh’s EPI provided additional ILRs on request to district and city corporation depots in preparation for the introduction of IPV. In our assessment, we found that depots at the national and subnational levels (districts and city corporations) had sufficient cold-chain storage capacity for vaccines, but a quarter of health facilities reported insufficient storage capacity. Although this insufficient cold-chain capacity did not stop immunization activities, future vaccine introductions in Bangladesh should include assessments of cold-chain storage capacity at lower levels (health facilities and outreach vaccination sites) to avoid further cold-chain gaps.

Vaccine transport is the most vulnerable component of the cold chain in Bangladesh, and maintaining the cold chain during transport requires further strengthening. WHO standards state that “cold life” (maintaining temperatures <10°C) of cold boxes with frozen ice packs should be 2–3 days [18]. In the current study, we found that some cold boxes transporting vaccines from subnational depots to health facilities reached temperatures >10°C after about 5 hours of transportation. Potential contributors might be placement of cold boxes in open trucks, where they were exposed to direct sunlight and high environmental temperatures, and filling cold boxes with too many vaccines and too few ice packs because of insufficient cold box capacity [14]. Increasing the number of cold boxes and ice packs used for transportation and changing ice packs during long distance trips could be feasible interventions. 

We also observed that temperatures reached <2°C and even below freezing in a few instances during transportation of vaccines in cold boxes and vaccine carriers. Periodic training of staff on simple measures such as conditioning ice packs before placement into cold boxes and protecting vaccine boxes from direct contact with frozen ice packs [7] could decrease the risk of exposure of vaccines to freezing during transportation.

Substantial vaccine wastage was found because IPV was provided in 5-dose vials, more than half (58%) of outreach vaccination sessions required only 1 or 2 doses per session, and current policy requires disposal of the open vials at the end of the vaccination session [11]. To ensure vaccine availability for the whole birth cohort, Bangladesh should consider implementing at all immunization sessions the 28-day open vial policy for IPV, which allows use of open vials of IPV for up to 28 days as long as the top of the vial is intact and the cold chain is maintained. This policy could substantially decrease the wastage of IPV. Implementation of this policy will require that open vials of IPV go back and forth from health facilities to outreach sessions in vaccine carriers. Currently, Bangladesh does not routinely include temperature monitors in cold boxes and vaccine carriers. As a result, occasional exposures of IPV to freezing temperatures found in this assessment may be causing damage to vaccine potency that is not being detected.

Although we observed instances of elevated temperatures in cold boxes and vaccine carriers of up to 21°C or lasting more than half a day, none of the VVMs were found to be in stage 3 or 4, suggesting low risk of vaccine potency loss in observed vials [19]. However, if the Bangladesh EPI implements the WHO-recommended 28-day open vial policy [20], the cumulative exposure of opened vials to elevated temperatures during more transport episodes can increase the risk of vaccine wastage. To prevent reduction in vaccine availability and potency, the Bangladesh EPI could examine interventions recommended by the WHO to facilitate close monitoring of temperatures during transport of vaccines to health facilities and to outreach sessions [18].

Our study has several limitations. We purposively selected areas for inclusion in the study, and as a result, our findings are not generalizable to the rest of Bangladesh. However, the selected areas represented a variety of settings in Bangladesh, including areas with high and low immunization coverage and urban, rural, and hard-to-reach areas, so that themes and issues that were either EPI system–wide or specific to a particular setting could be identified. The field teams also collected temperature measurements on a single day for a limited time. Additional episodes of temperature excursions would probably have been found with a longer period of observation or more monitoring events, strengthening the evidence to support the recommendation that more focus should be paid to temperature monitoring, especially during transport. Although this assessment could not evaluate the independent effects of IPV introduction on vaccine cold-chain capacity because PCV was introduced concurrently [3], certain findings, such as the need to use temperature monitoring during transport, were independent of the number of vaccines introduced.

Bangladesh’s cold chain demonstrated adaptability with the introduction of IPV (and PCV). The lessons learned from this assessment suggest that Bangladesh’s EPI and other countries planning to introduce IPV should pay special attention to ensuring consistent and sufficient cold-chain storage at the lower administrative levels and monitoring the cold chain during vaccine transportation at all levels.

## Notes

***Acknowledgments.*** We would like to acknowledge the following persons for their contribution to data collection: Kamrul Islam, Naheen Quiyum, Golam Mostafa, Tanvir Faruk, Mohammed Hafizullah, Muntasir Moin Nabil, Mohammed Ziaul Haque, Sadia Ashrafee, Arifon Nessa Jany, Nurul Islam Khan, and Mohammed Abdul Baten; we also acknowledge Habib Habib Abdullah Sohel, for his data management support. We thank all the families and EPI staff who generously provided their time to participate in the surveys. The icddr,b acknowledges with gratitude the commitment of the CDC to its research efforts and is also grateful to the governments of Bangladesh, Canada, Sweden, and the United Kingdom for providing core support.

***Financial support***. Funding for this study was supported by the Centers for Disease Control and Prevention, grant number GR- 00720.

***Supplement sponsorship***. This work is part of a supplement coordinated by the Task Force for Global Health with funding provided by The Bill and Melinda Gates Foundation and the Centers for Disease Control and Prevention.

***Potential conflicts of interest.*** All authors: No reported conflicts. All authors have submitted the ICMJE Form for Disclosure of Potential Conflicts of Interest. Conflicts that the editors consider relevant to the content of the manuscript have been disclosed.
